# AF‐FLOW Global Registry Confirms Validity of Electrographic Flow Mapping as a Phenotyping Tool for Atrial Fibrillation

**DOI:** 10.1111/jce.16568

**Published:** 2025-01-16

**Authors:** Kent R. Nilsson, Steven Castellano, Melissa H. Kong, Pawel Derejko, Tamás Szili‐Torok, Sandeep K. Goyal, Sip Wijchers, Mohit Turagam, Vivek Y. Reddy, Atul Verma, other members of the AF‐FLOW Global Registry Study Group, Kostiantyn Ahapov, Alexander Bardyszewski, Jacek Kuśnierz, Dobromila Dzwonkowska, Mark Hoogendijk

**Affiliations:** ^1^ Department of Cardiac Electrophysiology Piedmont Heart Institute Athens Georgia USA; ^2^ Augusta University‐University of Georgia Athens Georgia USA; ^3^ Cortex Inc. Menlo Park California USA; ^4^ Department of Cardiology Medicover Hospital Warsaw Warsaw Poland; ^5^ Department of Internal Medicine, Cardiology Center University of Szeged Szeged Hungary; ^6^ Department of Cardiology, Erasmus Medical Center Clinical Electrophysiology Unit Rotterdam the Netherlands; ^7^ Icahn School of Medicine at Mount Sinai New York New York USA; ^8^ McGill University Health Centre Montreal Canada

**Keywords:** atrial fibrillation, basket catheter, catheter ablation, electrographic flow mapping, panoramic mapping, PVI‐plus

## Abstract

**Background:**

Electrographic flow (EGF) mapping allows for the visualization of global atrial wavefront propagations. One mechanism of initiation and maintenance of atrial fibrillation (AF) is stimulation from EGF‐identified focal sources that serve as driver sites of fibrillatory conduction. Electrographic flow consistency (EGFC) further quantifies the concordance of observed wavefront patterns, indicating that a healthier substrate shows more organized wavefront propagation and higher EGFC. Freedom from AF (FFAF) recurrence has accordingly been shown to be higher in patients with ablated vs. unablated sources and with high vs. low EGFC.

**Objectives:**

(1) Measure FFAF across EGF‐derived phenotypes in patients enrolled in the *AF‐FLOW Global Registry*; (2) determine if a relationship exists between EGFC and percentage of healthy voltage as measured from bipolar voltage maps.

**Methods:**

The *AF‐FLOW Global Registry* is a multicenter, prospective study of 25 all‐comer AF patients who underwent concomitant high‐density bipolar voltage mapping with a 16‐electrode grid mapping catheter and EGF mapping with a 64‐pole basket catheter. The EGF algorithm detects extra‐pulmonary vein sources as origins of excitation from a singularity of divergent flow vectors and was used to localize RF ablation targets. Overall, EGFC per atrium was also computed as the average of the modulus of individual EGF vectors, where the vector length represents the consistency of flow patterns. Patients were then assigned phenotypes on the basis of source presence or absence and EGFC, and rates of FFAF at 1‐year were compared across the four resulting phenotypes. Atrial EGFC was also compared to the percentage of healthy tissue determined by bipolar voltage mapping.

**Results:**

Patients with paroxysmal AF had higher FFAF than persistent AF (PeAF) and long‐standing PeAF patients; patients receiving de novo ablation had higher FFAF than those receiving redo ablation. Patient phenotyping revealed that those with high EGFC had higher FFAF than those with low EGFC (*p* = 0.015). Atrial EGFC was also correlated to the percent of high voltage tissue across all patients (*r* = 0.651, *p* < 0.0001).

**Conclusions:**

EGF mapping provides insights into the mechanistic nature of AF and the atrial health of the underlying substrate. Therefore, further studies are needed to develop phenotype‐specific treatments for the disease.

**Trial Registration:**

ClinicalTrials.gov identifier: NCT05481359.

## 1 Introduction

Atrial fibrillation (AF) is a complex disease that can be triggered by a variety of mechanisms, including pulmonary vein (PV) triggers, extra‐PV triggers, micro‐ and macro‐reentry circuits, abnormal substrate, and epicardial fibers [1, 2, 3, 4, 5, 6]. Despite its multifactorial origins, PV isolation (PVI) remains the widely accepted gold standard treatment for both paroxysmal and persistent AF [7, 8]. However, approaches to improve ablation strategies beyond PVI have thus far focused largely on identifying an optimal set of lesions to apply to all patients rather than an individualized approach that targets the patient's specific underlying disease pathophysiology.

Electrographic flow (EGF) mapping has recently provided one potential means of understanding AF on a patient‐specific basis. EGF mapping allows for the visualization of electrical wavefront propagation, which enables physicians to both identify extra‐PV sources and characterize the consistency of wavefront patterns over time [9, 10]. The recent *FLOW‐AF* multicenter, randomized controlled trial of redo persistent AF (PeAF) and long‐standing PeAF (LS‐PeAF) demonstrated that patients had significantly higher freedom from AF (FFAF) recurrence at 12 months when extra‐PV sources were ablated vs. unablated and when electrographic flow consistency (EGFC), a measure of atrial substrate health, was high vs. low [11, 12, 13]. Four phenotypes were accordingly established as follows: patients with no sources and high EGFC had 100% FFAF; patients with sources and high EGFC had 88% FFAF when sources were ablated vs. 50% when unablated; patients with sources and low EGFC had 46% FFAF when sources were ablated vs. 23% when unablated; and patients with no sources and low EGFC had 50% FFAF. Furthermore, retrospective and prospective analyses have shown that 50%–70% of patients have such sources with women more likely to have both sources and high EGFC than men [14, 15].

Subsequently, the *FLOW EVAL‐AF* trial was performed to analyze EGF maps during both AF and sinus rhythm (SR) in a set of 10 patients mapped in both rhythms during a single procedure. Patients also received concurrent biatrial high‐density bipolar voltage maps in both rhythms. The study demonstrated that overall, EGFC was uniformly higher in SR compared to AF despite significant variations in flow propagation at specific anatomical sites between the two rhythms [16, 17]. These findings corroborated earlier rhythm‐dependent analyses in animal models [18]. In addition, the mean EGFC was found to be directly proportional to the percentage of healthy substrate measured by bipolar voltage mapping in both atria during both AF and SR [16, 17].

Here, we analyze the results of the *AF‐FLOW Global Registry* to build on previous findings related to EGF mapping, namely those from *FLOW‐AF* and *FLOW EVAL‐AF*. Using data from 25 all‐comer AF patients mapped in five centers across the United States and European Union, we evaluate the utility of patient phenotyping based on EGF‐identified sources and EGFC, and we determine if a larger relationship exists between EGFC and bipolar voltage.

## 2 Methods

### 2.1 Study Overview

All physicians had autonomy over how to integrate EGF mapping into their clinical procedures in the *AF‐FLOW Global Registry*. Twenty‐five patients were enrolled across five experienced, high‐volume centers in three countries. Patients were eligible if they were at least 18 years old with a history of documented, symptomatic AF and were candidates for catheter ablation. AF could be paroxysmal AF (PAF, i.e., no sustained episodes of more than 7 days), PeAF (i.e., sustained episode of AF lasting more than 7 days), or LS‐PeAF (i.e., sustained episode of AF lasting more than 1 year). Patients also consisted of both those presenting for redo ablation who previously received catheter ablation but had recurrent AF and those presenting for de novo ablation who were undergoing elective catheter ablation for the first time. Patients were excluded from the study if any of the following were present: pacemaker or other transvenous pacing/defibrillation leads; prosthetic mitral valve; known reversible causes of AF; any cerebral ischemic event (strokes or transient ischemic attacks) that occurred during the 6‐month interval preceding the consent date; history of thromboembolic event within the past 6 months or evidence of intracardiac thrombus at the time of the procedure; or they were unable to provide their own informed consent.

Patients first underwent high‐density bipolar voltage mapping using the 16‐pole grid mapping catheter, followed by baseline EGF mapping in their presenting rhythm: AF or SR. PVI was then performed in standard fashion, as detailed below. A 20‐minute waiting period to confirm electrical isolation of the PVs was required, and PV reconnections were ablated when necessary. If patients were not already in AF, AF was induced after completion of PVI. EGF mapping then proceeded in AF. If AF was not inducible, mapping and EGFC calculation proceeded in SR.

All patients were evaluated 7 days post‐procedure with a follow‐up phone call to document any adverse events and/or any medication changes. Mandatory ambulatory monitoring was performed at 12 months post‐procedure. Unscheduled visits for symptomatic AF or adverse events were additionally tracked. If any AF was detected by EKG or Holter monitoring during any scheduled ambulatory monitoring or unscheduled visit outside a 3‐month post‐procedure blanking period, patients were considered to have AF recurrence after their EGF‐mapping procedure. If no such events occurred, patients were considered to have FFAF. Patients who did not complete 12‐month follow‐up and who otherwise did not have documented recurrence outside the blanking period were considered lost to follow‐up (LTFU).

The trial was sponsored by Cortex Inc. (formerly Ablacon Inc.) and approved by the appropriate Ethics Committees at each of the participating centers. Data monitoring, collection, and analysis were performed by the sponsor using a third‐party clinical research organization. The authors assume responsibility for the accuracy and completeness of the data.

### 2.2 Ablation Procedure

All procedures were performed under sedation after obtaining informed consent. Patients underwent three‐dimensional electroanatomic mapping with either Ensite NavX (Abbott, Abbott Park, IL) or CARTO (Biosense Webster, Diamond Bar, CA), followed by PVI using an irrigated tip radiofrequency (RF) ablation catheter. Ablation catheter power and temperature settings were at the operator's discretion. Intravenous heparin was administered for systemic anticoagulation to maintain an activated clotting time of > 300 s before insertion of the 64‐pole basket mapping catheter. For any ablation of PVs, confirmation of PVI (entrance or exit block) was performed after a mandatory minimum 20‐min wait following the last RF application.

### 2.3 EGF Mapping

EGF mapping was performed both pre‐ and post‐PVI using the Ablamap v11.1.0.56 software (Ablacon, Wheat Ridge, CO). Unipolar electrograms were recorded using a commercially available 64‐electrode basket mapping catheter connected to a proprietary, CE‐marked recording system (EP Map, Herdecke, Germany). Each recording was 1‐min long, and the unipolar signals were processed using a proprietary algorithm to remove the QRS complex, noise, baseline fluctuations, and far‐field signals. Using a biharmonic spline interpolation based on Green's function, the electrical field was estimated, and a Horn–Schunck flow estimation of the Green's interpolation frames was performed to visualize atrial wavefront propagation over time. Haines et al. published an in‐depth explanation of the theoretical basis for EGF mapping and details of the EGF mapping algorithm [9]. The dominant patterns of wavefront propagation over time are displayed as flow vectors in EGF Segment Maps, and the consistency of these flow vectors is measured over time, enabling the visualization of origins of excitation representing extra‐PV AF triggers, called sources. Active sources exhibit divergent flow fields.

**Figure 1:**
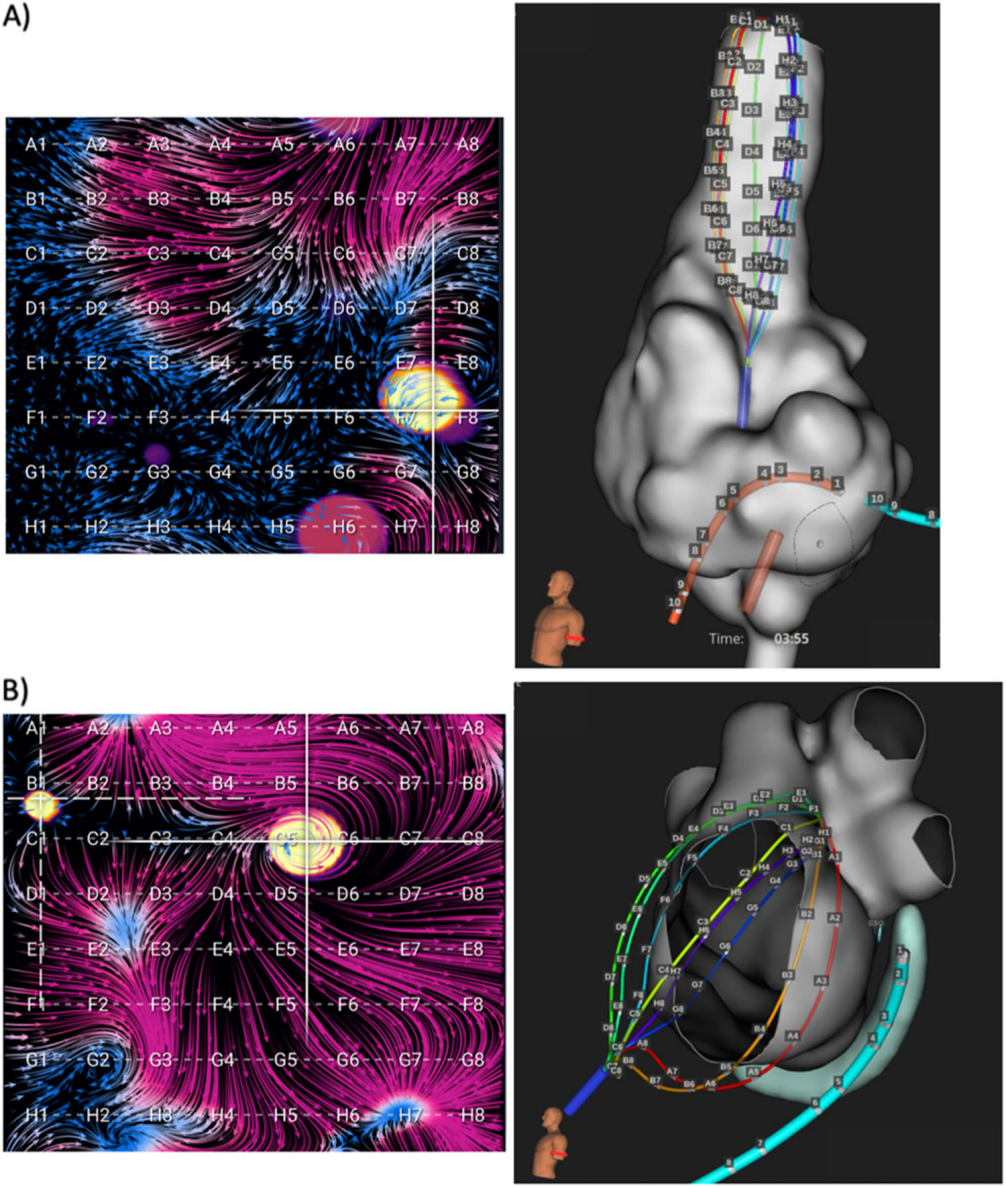
Post‐PVI EGF maps and accompanying electroanatomic maps. Mapping in (A) SVC/RA junction (RA position 1) and (B) posterosuperior LA (LA position 1) are shown. Note that the RA map has a larger surface area with low EGFC (seen as shorter vectors in blue), resulting in an EGFC for the map of 0.51, while the LA map has more areas containing high EGFC (seen as longer vectors in purple), resulting in an EGFC for the map of 1.21. An active source can be seen in the RA at E7‐F8 (crosshairs) with a SAC of 25%, while the LA source at C5‐C6 has 37% SAC.

EGF mapping was generally performed by obtaining 1‐min recordings from the 64‐pole basket catheter placed in multiple standardized positions within each atrium as well as additional recordings after any source ablations to confirm elimination. In the left atrium (LA), there were typically two standardized basket positions: (1) posterosuperior LA with the dome of the basket aiming toward the left PVs and LA appendage (LAA); (2) lateral LA with basket dome pointing toward LAA and the mitral valve. In the right atrium (RA), there were generally three standardized basket positions: (1) superior vena cava (SVC)/RA junction with basket position at least halfway into the SVC; (2) central RA with basket dome pointing to SVC with vertical catheter shaft orientation and basket expanded to fill RA; (3) anterolateral RA with basket dome pointing to RA appendage and tricuspid valve and catheter deflected anterior and lateral from SVC. Mapping in additional basket positions in either atrium was performed if necessary to obtain complete endocardial atrial coverage. Example EGF maps in each atrium with accompanying electroanatomic maps are shown in Figure 1.

Sources were defined as origins of divergent flow with source activity (SAC) quantified as the percentage of 2 s segments within a recording during which the source was emanating flow. If EGF mapping identified an extra‐PV source with a SAC ≥ 25%, which was the prespecified threshold for clinical significance, EGF‐guided ablation at the target location of the focal source was advised. Repeat EGF mapping was recommended to confirm the elimination of the source via reduction of SAC below 25%. If additional sources were unmasked during repeat mapping, additional ablation was advised until all sources were eliminated.

### 2.4 Near‐Field Score Calculations From EGM Traces

To estimate the contribution of flow and far‐field to the signal, we assumed that signal energy could be decomposed into conduction, instantaneous, and fractionation components:

$$E_{\mathrm{signal}}=E_{\mathrm{cond}}+E_{\mathrm{inst}}+E_{\mathrm{frac}}$$

Energy ratios of the components could then be defined as follows:

$${\mathrm{RE}}_{\mathrm{cond}}=\frac{E_{\mathrm{cond}}}{E_{\mathrm{signal}}},\;{\mathrm{RE}}_{\mathrm{inst}}=\frac{E_{\mathrm{inst}}}{E_{\mathrm{signal}}},\;{\mathrm{RE}}_{\mathrm{frac}}=\frac{E_{\mathrm{frac}}}{E_{\mathrm{signal}}}$$

where:

$${\mathrm{RE}}_{\mathrm{cond}}+{\mathrm{RE}}_{\mathrm{inst}}+{\mathrm{RE}}_{\mathrm{frac}}=1$$

To compute the conduction component, neighboring channels of all electrodes were first determined. In so doing, the 64‐electrode basket mapping catheter EGM channels were rearranged into an 8 × 8 grid such that splines became rows A, B, …, H and electrode rings became columns 1, 2, …, 8. For every EGM channel, four neighbors were then extracted. For channels on the A and H splines, channels with the same column number on the opposite spline were considered neighbors to account for the circular boundary. Lastly, to account for polar boundary conditions, artificial polar electrodes were computed as averages of electrodes A1, B1, …, H1 and A8, B8, …, H8 as neighbors to A1, B1, …, H1 and A8, B8, …, H8, respectively.

Normalized cross‐correlation between every channel and all neighbors was then computed according to the equation below, where *X* and *Y* are two neighboring channels, N=len(X)/2, and ${\left[X\times Y\right]}_{\mathrm{dt}}$ was the cross‐correlation with offset dt ms:

$${\left[X\times Y\right]}_{\mathrm{dt}}=\frac{\sum_{\mathrm{dt}=1}^{N}X_{t+\mathrm{dt}}Y_{t}}{\sqrt{\sum_{t+1}^{N}{X[t]}^{2}\sum_{t=1}^{N}{Y[t]}^{2}}}$$

The conduction component between two signals was determined by the highest peak in the normalized cross‐correlation between these signals. Notably, the conduction component was 0 if dt>50 or dt<5ms because correlation is not attributed to conduction when it is respectively too slow or too fast for conduction. The conduction component of the channel was accordingly defined as the mean of non‐zero conduction components between the channel and its neighbors:

$${\mathrm{RE}}_{\mathrm{cond}}\left(X,Y\right)=\max_{\mathrm{dt}=1}^{N}{\left[X\times Y\right]}_{\mathrm{dt}}$$

$$\mathrm{dt}\prime ={\mathrm{argmax}}_{\mathrm{dt}=1}^{N}{\left[X\times Y\right]}_{\mathrm{dt}}$$

$${\mathrm{RE}}_{\mathrm{cond}}\left(X\right)=\mathrm{mean}\{Y\;\mathrm{is}\;a\;\mathrm{neighbor}\;\mathrm{of}\;X,\;{\mathrm{RE}}_{\mathrm{cond}}\left(X,Y\right)\neq 0\}\;{\mathrm{RE}}_{\mathrm{cond}}\left(X,Y\right)$$

In case the conduction component equaled 0, the instantaneous component between signals picked up by two neighboring channels was estimated as the normalized correlation at dt=0. The instantaneous component of an individual channel was then estimated as an average of instantaneous components between the channel and all neighbors:

$${\mathrm{RE}}_{\mathrm{inst}}\left(X\right)=0\;\text{if}{\mathrm{RE}}_{\mathrm{cond}}\left(X\right)\neq 0$$

$${\mathrm{RE}}_{\mathrm{inst}}\left(X\right)=\mathrm{mean}\;\{Y\;\mathrm{is}\;a\;\mathrm{neighbor}\;\mathrm{of}\;X\}\;\;{\left[X\times Y\right]}_{0}\;\text{if}\;{\mathrm{RE}}_{\mathrm{cond}}\left(X\right)=0\;$$

**Figure 2:**
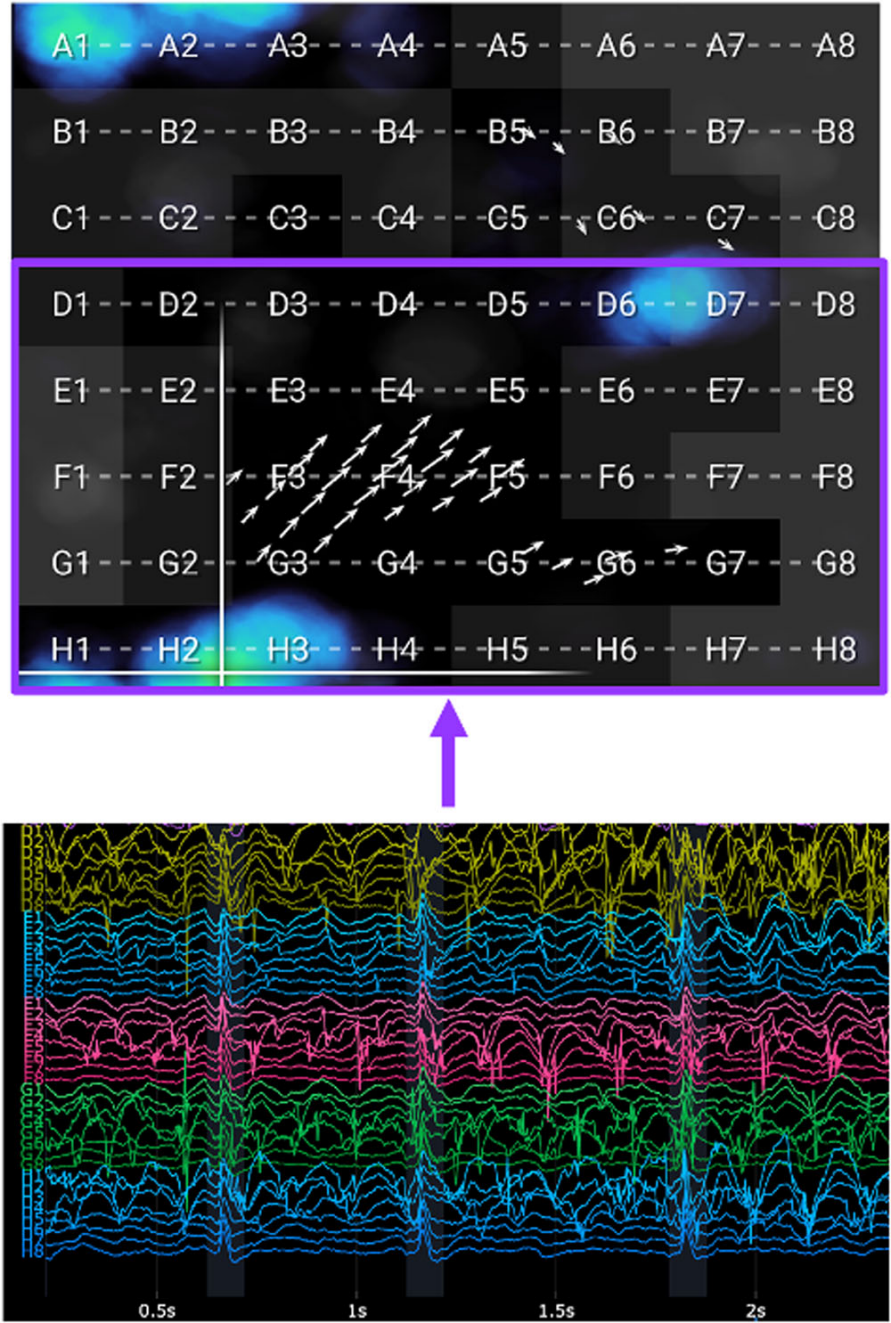
Near‐field score calculation. (Top) An EGF summary map with grey squares representing electrodes with near‐field scores < 0.7. Near‐field score was calculated as the percentage of segments with cross‐correlation > 0 with at least one of the adjacent channels at a time shift between 5 and 50 ms. Accordingly, grey electrodes had cross‐correlation with adjacent channels in < 70% of the segments. Because a low near‐field score was a proxy for poor atrial contact, these electrodes were not considered in EGFC calculations from maps. (Bottom) The first 2 s of the recording from channels in splines D–H. Channels corresponding to grey electrodes, including the eighth electrode in all splines, did not have depolarizations correlating to those in any neighboring channels at a time offset between 5 and 50 ms, and therefore had a near‐field score < 0.7.

The near‐field score per segment was ultimately defined as a binary mask: 1 if ${\mathrm{RE}}_{\mathrm{inst}}$ < 0.3 and 0 otherwise. All computations were performed by segment, so aggregation for the whole recording was lastly done by averaging along the segment axis. Thus, the summative near‐field score per electrode showed the percentage of segments at a given location when the near‐field score equaled 1. Contact was considered poor when the summative near‐field score was < 0.7. An example EGF summary map with constituent EGMs from electrodes above and below this near‐field score threshold is shown in Figure 2.

### 2.5 EGFC Calculations From EGF Maps

EGFC was computed for each recording from the Euclidean length of vector field estimates across all basket electrodes over 60 s of recording time, thereby providing a measure of the overall magnitude of flow averaged in arbitrary units. Importantly, electrodes with low contact—defined as a near‐field score < 0.7—were removed from the calculation because of artificially low local EGFC values that were observed at non‐contacting electrode positions.

After calculating EGFC for each recording, an overall EGFC was also computed for each patient in either AF or SR. The EGFCs from each recording position during a given rhythm were first averaged to compute positional EGFC means per patient. These positional EGFCs were then averaged within each atrium to generate a mean EGFC per atrium per patient. Such a method allowed all recording positions to be given equal weight.

Each patient's two atrial EGFCs were then averaged again to generate an overall mean EGFC per patient. When generated from AF recordings, this overall mean EGFC was employed in phenotyping. When no AF recordings were available, the mean EGFC was computed in SR and converted to an AF EGFC using a linear equation developed previously [16, 17].

### 2.6 Phenotyping

Patients were phenotyped based on EGF map properties. Type I patients had SAC < 25% in all recordings and EGFC overall mean ≥ EGFC median of all patients; Type II had active sources with SAC ≥ 25% and EGFC overall mean ≥ EGFC median of all patients; Type III had active sources with SAC ≥ 25% and low EGFC < EGFC median; and Type IV had SAC < 25% and EGFC overall mean < EGFC median.

### 2.7 Voltage Map Analysis

Bipolar voltage maps were collected with standardized mapping parameters so that voltages greater than 0.5 mV were purple, voltages less than 0.1 mV were grey, and intermediate voltages ranged from blue to red as voltage decreased. Accordingly, a color detector was built using OpenCV 4.7.0 in Python 3.12 to quantify the number of pixels in each color. The fraction of purple pixels divided by all other colored pixels was then calculated to determine the percentage of high voltage surface area in each voltage map. The threshold for high vs. low voltage was deemed to be 0.5 mV in accordance with it being the established cutoff regarded in the literature [16, 17, 19, 20, 21]. The high voltage percent was then plotted against the mean atrial EGFC that had been calculated contemporaneously from the same atrium of the same patient in the same rhythm.

### 2.8 Statistical Analysis

Summary data are expressed as mean value ± one standard deviation. *p* values for group comparisons were computed using one‐tailed, independent *t*‐tests for continuous variables and one‐tailed *z*‐tests for proportions, as appropriate. Linear regression was calculated using a line of best fit, and the coefficient of determination (*r* ^2^) and the *p* value for the *F*‐test of significance of the slope were reported. For all statistical tests, the null hypothesis was rejected at the level of *p* < 0.05.

## 3 Results

### 3.1 Patient and Procedure Characteristics

A total of 25 patients were enrolled with a mean age of 64 ± 11 years. Nine (36%) patients were female. Mean LA diameter was 4.4 ± 0.8 cm, and mean left ventricular ejection fraction was 57% ± 8%. Sixteen (64%) patients were de novo ablations, while 10 (36%) were redo. Nine (36%) had PAF, 14 (56%) had PeAF, and two (8%) had LS‐PeAF. Baseline patient characteristics are summarized in Table 1. The PAF group had significantly fewer patients with history of prior ablation (2/9 vs. 13/16, *p* = 0.002) and history of cardioversion (2/9 vs. 12/16, *p* = 0.005).

**Table 1:** Patient characteristics.

	All (*n* = 25)	PAF (*n* = 9)	PeAF and LS‐PeAF (*n* = 16)
**Demographics**			
Age, mean ± SD	63.9 ± 11.2	67.8 ± 10.9	61.8 ± 11.5
Female, *n* (%)	9/25 (36%)	5/9 (56%)	4/16 (25%)
BMI, mean ± SD	27.9 ± 3.9	27.7 ± 5.0	28.0 ± 3.3
**Patient risk factors**			
History of smoking, *n* (%)	7/25 (28%)	4/9 (44%)	3/16 (19%)
CHA_2_DS_2_VASc score, mean ± SD	2.7 ± 1.9	2.8 ± 1.6	2.7 ± 2.1
Diabetes, *n* (%)	4/25 (16%)	1/9 (11%)	3/16 (19%)
Hypertension	16/25 (64%)	6/9 (67%)	10/16 (63%)
LA diameter (cm), median [IQR]	4.4 [3.8–4.8]	4.0 [3.8–4.4]	4.7 [4.1–5.1]
LA volume index (mL/m^2^), median [IQR]	40 [29–52]	28 [23–33]	47 [36–53]
LVEF (%), median [IQR]	55 [55–60]	56.5 [55–60]	55 [55–60]
**Patient history**			
History of prior ablation, *n* (%)	15/25 (60%)	2/9 (22%)	13/16 (81%)
History of AFL, *n* (%)	6/23 (26%)	3/9 (33%)	3/14 (21%)
History of cardioversion, *n* (%)	14/25 (56%)	2/9 (22%)	12/16 (75%)
Number of past cardioversions of cardioverted patients, mean ± SD	1.9 ± 0.9	1.5 ± 0.7	1.9 ± 1.0

**Figure 3:**
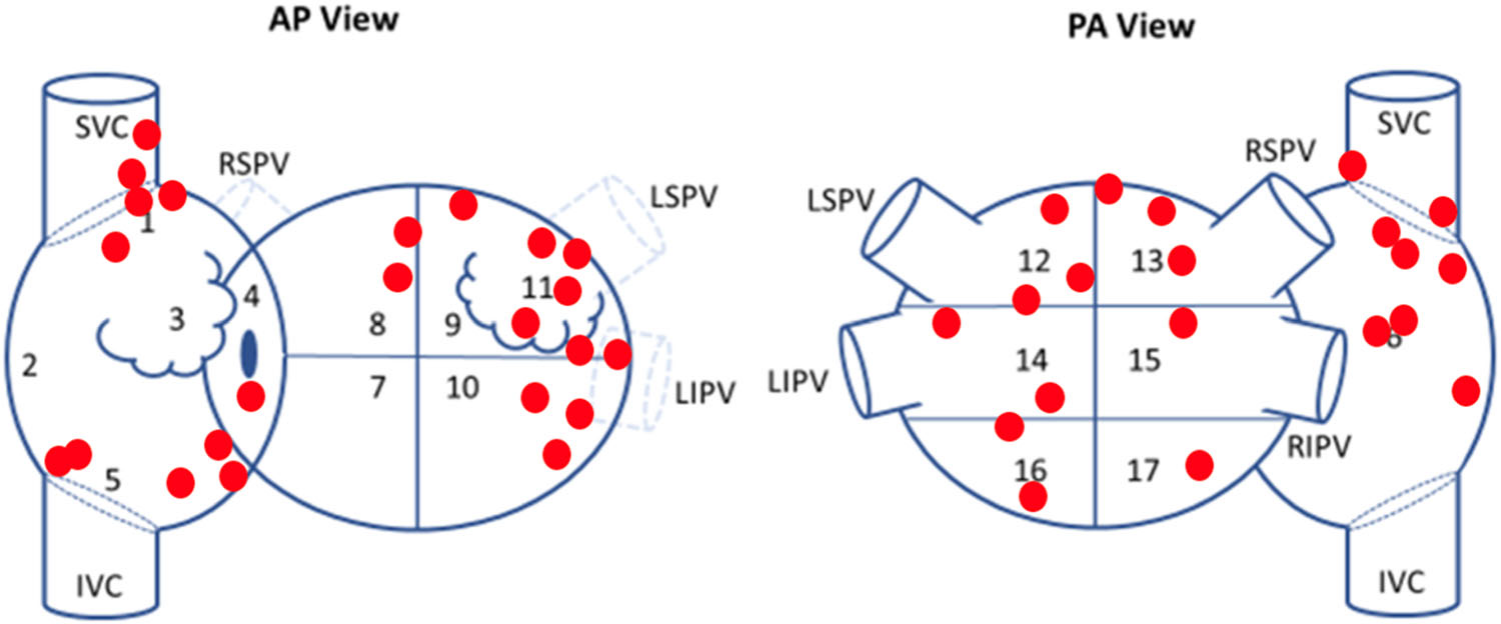
Anatomic distribution of EGF‐identified sources. Source locations are shown in red on a schematic of the left and right atria. AP, anteroposterior; IVC, inferior vena cava; LIPV, left inferior pulmonary vein; LSPV, left superior pulmonary vein; PA, posteroanterior; RIPV, right inferior pulmonary vein; RSPV, right superior pulmonary vein; SVC, superior vena cava.

All patients received PVI and had isolation confirmed after at least a 20‐min waiting period. At the time of PVI, 13 (52%) patients were in spontaneous AF, and 12 (48%) required AF induction with rapid or decremental atrial burst pacing. After PVI, 6 (24%) patients were non‐inducible. Overall, 43 total sources were identified by EGF mapping in the anatomic distribution shown in Figure 3. At least one extra‐PV source was found in 64% (16/25) of patients, 81% (13/16) of whom received at least one EGF‐guided ablation at the physician's discretion. However, only 19% (3/16) had all EGF‐identified sources with SAC ≥ 25% ablated. These 3 patients and the additional 9 patients with no extra‐PV sources were therefore considered to be treated optimally per protocol, while the remaining 13 (52%) patients were not. Procedural characteristics are summarized in Table 2. PAF patients presented less often in AF (0/9 vs. 13/16, *p* < 0.001) and were more often non‐inducible (6/9 vs. 0/16, *p* < 0.001). They accordingly had fewer EGF‐identified sources (0.9 ± 1.2 vs. 2.2 ± 1.3, *p* = 0.011) and were more often treated per protocol (7/9 vs. 5/16, *p* = 0.013).

**Table 2:** Procedural characteristics.

Cohort	All (*n* = 25)	PAF (*n* = 9)	PeAF and LS‐PeAF (*n* = 16)
Presented in AF, *n* (%)	13/25 (52%)	0/9 (0%)	13/16 (81%)
Non‐inducible post‐PVI, *n* (%)	6/25 (24%)	6/9 (67%)	0/16 (0%)
Source(s) detected in patient, *n* (%)	16/25 (64%)	3/9 (33%)	13/16 (81%)
Sources per patient, mean ± SD	1.7 ± 1.4	0.9 ± 1.2	2.2 ± 1.3
Any source ablation, fraction (%)	13/16 (81%)	3/3 (100%)	10/13 (77%)
All source ablation, fraction (%)	3/16 (19%)	1/3 (33%)	2/14 (14%)
Treated per protocol, *n* (%)	12/25 (48%)	7/9 (78%)	5/16 (31%)
Total ablation time of ablated patients (min), mean ± SD	3.59 ± 0.94	3.27 ± 1.11	3.78 ± 0.81

### 3.2 Clinical Outcomes Provide Support for EGF‐Mapping as an AF Phenotyping Tool

Twenty‐one (84%) patients completed 12‐month follow‐up, and 13 (62%) had FFAF while 9 (38%) had documented recurrence. When broken down by AF type, 6/8 (75%) PAF patients had FFAF, while 5/11 (45%) PeAF patients and 1/2 (50%) LS‐PeAF patients had FFAF. Collectively, PAF vs. PeAF and LS‐PeAF recurrences were not significantly different (*p* = 0.10). However, when analyzed by procedure type, 11/13 (85%) de novo patients had FFAF, while 2/8 (25%) redo patients had FFAF (*p* = 0.003). Accordingly, there was significantly higher FFAF in de novo PAF vs. redo PAF patients (5/5 vs. 1/3, *p* = 0.017) and significantly higher FFAF in de novo PeAF and LS‐PeAF vs. redo PeAF and LS‐PeAF patients (6/8 vs. 1/5, *p* = 0.026). There were no significant differences observed in post‐procedure outcomes in men vs. women.

The median global EGFC across all patients was 0.548. When patients who completed 12‐month follow‐up were split such that those with global EGFC greater than the median were compared to those with global EGFC less than the median, the high EGFC patients had significantly higher FFAF than those with low EGFC (9/10 vs. 5/11, *p* = 0.015). EGFC and source presence could then be collectively incorporated to split the patients by phenotype, as shown in Table 3. FFAF decreased with increasing phenotype from Type I (high EGFC, no sources) with 3/3 (100%) FFAF to Type II (high EGFC, sources) with 6/7 (86%) FFAF to Type III (low EGFC, sources) with 4/7 (43%) FFAF to Type IV (low EGFC, no sources) with 1/4 (25%) FFAF.

**Table 3:** Results by phenotype.

**Results by phenotype (*n*** = **25)**				
Phenotype	Type I	Type II	Type III	Type IV
Total, *n* (%)	4/25 (16%)	8/25 (32%)	8/25 (32%)	5/25 (20%)
De novo procedures, *n* (%)	4/4 (100%)	5/8 (63%)	5/8 (63%)	2/5 (40%)
Paroxysmal, *n* (%)	3/4 (75%)	2/8 (25%)	1/8 (13%)	2/5 (40%)
EGFC (AUs), mean ± SD	0.64 ± 0.06	0.74 ± 0.20	0.44 ± 0.10	0.44 ± 0.07
Sources per patient, mean ± SD	0 ± 0	2.6 ± 1.9	2.6 ± 1.5	0 ± 0
Received EGF‐guided ablation, *n* (%)	0 (0%)	6/8 (75%)	7/8 (88%)	0 (0%)
Completed follow‐up, *n* (%)	3/4 (75%)	7/8 (88%)	7/8 (88%)	4/5 (80%)
All FFAF, *n* (%)	3/3 (100%)	6/7 (86%)	4/7 (43%)	1/4 (25%)
Per protocol FFAF, *n* (%)	3/3 (100%)	1/1 (100%)	1/1 (100%)	1/4 (25%)

**Figure 4:**
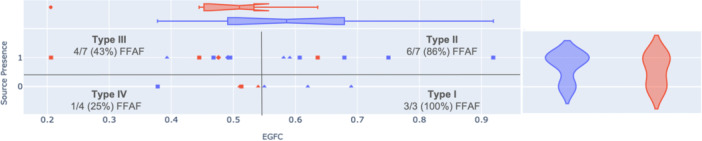
FFAF by source presence and EGFC. Patients with zero sources were considered to have no sources, while patients with one or more sources were considered to have sources. Most but not all sources were ablated during the procedure. The median EGFC across all 25 patients was 0.548, so was defined as the dividing line for high vs. low EGFC in this study. FFAF patients are shown in blue, and recurrence patients are shown in red; PAF is shown as triangles, PeAF is shown as squares, and LS‐PeAF is shown as diamonds. FFAF goes down with progressively increasing phenotype from Type I to Type IV.

Source presence vs. EGFC is plotted and divided by phenotype in Figure 4. Notably, most patients with sources had some, but not all, sources ablated, so they were not treated per protocol. Of the three patients who had all EGF‐identified sources ablated, 1 was a Type II patient who had FFAF, 1 was a Type III patient who had FFAF, and 1 was a Type III patient who was LTFU.

Patient phenotype was also related to patient AF status and procedure type. As shown in Figure 4, PAF patients were more likely to be Type I than PeAF and LS‐PeAF patients (3/9 vs. 1/16, *p* = 0.038) with other phenotypes not significantly different by AF type. De novo percentage also decreased with increasing phenotype from Type I with 4/4 (100%) de novo procedures to Types II and III each with 5/8 (63%) de novo procedures to Type IV with 2/5 (40%) de novo procedures.

### 3.3 EGFC is Correlated to Bipolar Voltage

**Figure 5:**
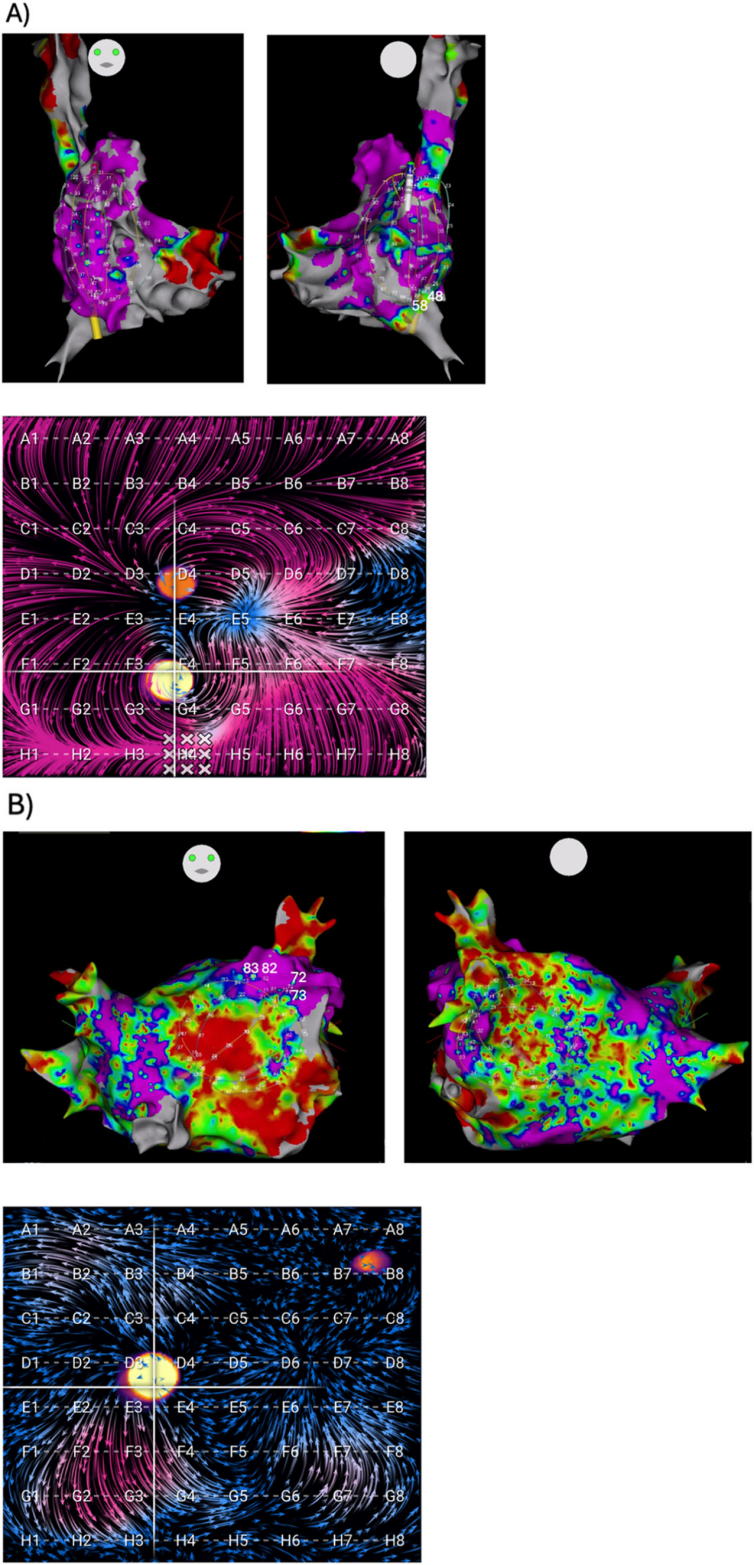
Local EGFC alignment with bipolar voltage. EGF maps and accompanying voltage maps from a single patient in AF are shown in the (A) RA and (B) LA. Areas of high EGFC (magenta) correspond to areas of high voltage (purple), while areas of low EGFC (blue) correspond to areas of low voltage (red). In the RA, all splines pass predominantly through high voltage except for electrodes 48–58 (D8‐E8 on EGF map). In the LA, all splines pass predominantly through medium and low voltage except for electrodes 72–83 (F2‐G3 on EGF map).

Across all patients, EGFC was related to bipolar voltage. Visually, bipolar voltages aligned with EGFC in both atria in both AF and SR. Following the splines of the basket catheter across the EGF maps and voltage maps particularly revealed similar trends in bipolar voltage and EGFC at the same locations. As shown in Figure 5, both overall trends in map EGFC and voltage patterns, as well as specific electrode‐by‐electrode alignment, corresponded closely between the two metrics. Bipolar voltage and EGFC were also both higher in SR than AF, such that percent healthy voltage was 52% ± 15% vs. 44% ± 11% (*p* = 0.042) and EGFC was 0.69 ± 0.21 vs. 0.57 ± 0.21 (*p* = 0.045). There was no significant difference between percent healthy voltage or EGFC in the RA vs. LA.

**Figure 6:**
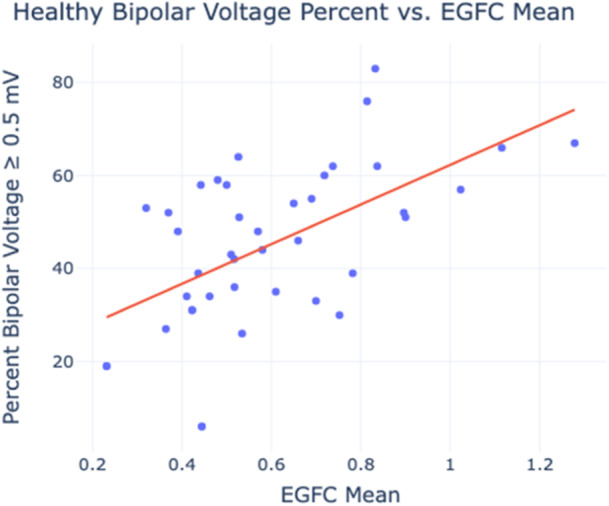
Global EGFC correlation to healthy bipolar voltage. Scatter plot of percent healthy voltage surface area vs. atrial EGFC from concomitant voltage and EGF maps. A best‐fit trendline shows a positive correlation (*r* = 0.651, *p* < 0.001).

To quantify the relationship between EGFC and voltage, bipolar voltage from the voltage maps was compared to global EGFC. The percentage of high voltage surface area across each atrium in each rhythm was plotted against the mean EGFC of the concomitant EGF maps recorded from the same atria in the same rhythm. As shown in Figure 6, healthy voltage percent increased linearly with EGFC (*r* = 0.651, *p* < 0.001) in the analyzed zone of healthy voltage percentages between 30% and 70% and EGFC between 0.3 and 1.2. This relationship was maintained across SR and AF in both the LA and RA.

## 4 Discussion

The *AF‐FLOW Global Registry* represents the first attempt to track the usage of EGF mapping in an open registry across a wide cohort of PAF, PeAF, and LS‐PeAF patients. Two major conclusions can be drawn:

1 Patients with high EGFC (Types I and II) have higher rates of FFAF than those with low EGFC (Types III and IV) among a diverse set of AF patients from multiple clinical centers receiving a variety of treatment modalities.
2 Atrial EGFC is correlated to atrial bipolar voltage across this same patient population.

The patient sample under study is broader than the redo PeAF and LS‐PeAF‐only population previously analyzed when phenotyping patients in *FLOW‐AF* [12]. The study also contains a larger number of patients across multiple clinical centers than *EVAL‐AF* but documents a similar relationship between EGFC and bipolar voltage [16, 17]. Therefore, although the *AF‐FLOW Global Registry* lacks internal controls and consists of only 25 patients, it demonstrates that the findings in *FLOW‐AF* and *EVAL FLOW‐AF* are robust in a new, broader population and that the benefits of EGF mapping still apply in real‐world settings where physicians use it as a primary or supplemental tool to their standard workflow.

Phenotyping continued to be a valuable predictive tool, most notably as high EGFC was related to higher rates of FFAF. In addition, Type I patients with no extra‐PV sources and high EGFC continued to have 100% FFAF from PVI‐only procedures, as was initially demonstrated in *FLOW‐AF* wherein 10/10 Type I patients did not recur [12]. Notably, phenotype was more predictive of outcome than AF type because outcomes were not significantly improved in PAF patients despite the lower rates of prior ablations and cardioversions, higher rates of presentation in SR and non‐inducibility, and lower source counts in PAF patients. Still, the *AF‐FLOW Global Registry* did establish that Type I patients were more likely to have PAF and to be receiving de novo ablation, signifying that the lack of pathophysiological markers of disease aligns with clinical observations suggestive of an earlier disease state.

The source axis of phenotyping was less meaningful in the *AF‐FLOW Global Registry* than EGFC because patient sources were largely neither strictly fully ablated nor left fully unablated. Many received PVI plus EGF‐guided source ablation, but few received complete ablation of all EGF‐identified sources per protocol. Accordingly, the FFAF for the mixed PAF and PeAF population was lower in this study than in the treatment population of redo PeAF and LS‐PeAF patients in *FLOW‐AF* [12]. Still, it should be noted that all patients who received complete source ablation per protocol in the *AF‐FLOW Global Registry* had no documented recurrences.

The demonstrated relationship of EGFC to bipolar voltage provides further credence to the hypothesis that EGFC is a measure of atrial substrate health. In addition, it implicates EGF mapping as a valuable tool for acquiring substrate information because EGF maps can be acquired faster than voltage maps with only 1 min of recording necessary per basket mapping position. Moreover, EGF mapping allows source information to be concurrently obtained. The substrate information from EGFC can accordingly be used to guide post‐procedure care, such as the initiation or continuation of antiarrhythmic drug therapies. Still, additional studies are needed to further define EGFC's role in the long‐term management of AF post‐ablation. Integration of EGF mapping with electroanatomic mapping is also necessary to more easily localize areas of high vs. low substrate health.

In the future, strategies to diagnose a patient's AF by pathophysiology and to provide treatment based on these AF phenotypes will continue to expand. As more EGF mapping data becomes available, we are pursuing efforts to mechanistically subtype identified sources as focal, macro‐reentrant, or micro‐reentrant and to tune ablation strategies to these mechanisms. Specialized approaches are also being designed for treating Type IV patients who have no extra‐PV sources but poor substrate, as PVI‐only has been insufficient in these patients. Type I patients with no extra‐PV sources and healthy substrate will also continue to be studied as a group that responds especially well to PVI‐only procedures.

### 4.1 Limitations

The major limitation of this study is the lack of a fixed protocol for all patients because the study was an open registry, which resulted in physicians not fully ablating all sources visualized. This limitation was compounded by the low number of patients enrolled. The ongoing *RESOLVE‐AF* trial is designed to rectify these limitations by analyzing and phenotyping a larger sample of over 200 redo and de novo patients for the purpose of phenotyping and measuring outcomes. Another limitation of this study is that during the alignment of EGF maps and bipolar voltage maps, the sampling of tissue was not equal across both mapping systems. Some regions of the atria were EGF mapped multiple times as the catheters were moved into the standardized positions, and other areas (e.g., the PVs beyond the ostia) were not mapped at all. Bipolar voltage mapping similarly did not always cover the full atria and was impacted by the orientation of the mapping catheter. Despite this limitation, a robust relationship between bipolar voltage and EGFC was still found.

## 5 Conclusion

The *AF‐FLOW Global Registry* consisted of 25 patients from five clinical centers with a variety of AF presentations and treatment strategies augmented by EGF mapping. Across all patients, high EGFC was most predictive of FFAF at 1‐year, while low EGFC was most predictive of recurrence. Mean EGFC was also correlated with the percent of high‐voltage tissue in each atrium across multiple rhythms. Further investigation of phenotyping will be conducted in the ongoing *RESOLVE‐AF* trial.

## AF‐FLOW Global Registry Study Group

**Kostiantyn Ahapov**, Cortex Inc., Menlo Park, California, USA; **Alexander Bardyszewski**, Department of Cardiology, Medicover Hospital Warsaw, Warsaw, Poland; **Jacek Kuśnierz**, Department of Cardiology, Medicover Hospital Warsaw, Warsaw, Poland; **Dobromila Dzwonkowska**, Department of Cardiology, Medicover Hospital Warsaw, Warsaw, Poland; **Mark Hoogendijk**, Department of Cardiology, Erasmus Medical Center, Clinical Electrophysiology Unit, Rotterdam, the Netherlands.

## Ethics Statement

The study protocol was reviewed and approved by the Ethics Committees at all participating medical centers.

## Consent

All patients gave written informed consent per IRB guidelines.

## Conflicts of Interest

Steven Castellano is an employee of and owns equity in Cortex Inc. Melissa H. Kong is a former employee of Cortex Inc., who owns equity and has patent holdings. Vivek Y. Reddy and Atul Verma are members of Cortex's scientific advisory board, who own equity. Tamás Szili‐Torok consulted for product development with Cortex Inc. The other authors declare no conflicts of interest.

## Data Availability

The data that support the findings of this study are openly available in clinicaltrials.gov, reference number NCT05481359: https://clinicaltrials.gov/study/NCT05481359?cond=atrial%20fibrillation&intr=egf%20mapping&rank=4.
